# Empowering youth and ensuring health: utilization of youth friendly service among preparatory school students in Gambella, Southwest Ethiopia

**DOI:** 10.3389/frph.2024.1452315

**Published:** 2024-12-11

**Authors:** Nardos Hailu, Benti Negero, Keno Melkamu, Yawkal Tsega

**Affiliations:** ^1^ Department of Maternal and Child Health, Gambella Primary Hospital, Gambella Regional Health Bureau, Gambella, Ethiopia; ^2^ Department of Public Health, College of Health Sciences, Mettu University, Mettu, Ethiopia; ^3^ Department of Health Systems and Management, School of Public Health, College of Medicine and Health Sciences, Wollo University, Dessie, Ethiopia

**Keywords:** youth friendly service, utilization, Gambella town, Southwest, Ethiopia, 2023

## Abstract

**Background:**

Youth is a period with exposure to high risk of reproductive health (RH) problems. Despite, several strategies designed to solve these problems, youths are experiencing unsafe abortion, unintended pregnancy, and sexually transmitted infections (STIs) so far. The utilization of Youth Friendly Services (YFS) and its determinants has not been well studied in Gambella so far. This study aimed to assess YFS utilization and associated factors among preparatory school students in Gambella town, Southwest Ethiopia.

**Methods:**

Institution based cross-sectional study was conducted on 394 randomly selected preparatory school students in Gambella town from June 1-30/2023. Data were collected through self-administered questionnaire, entered to EpiData version 4.6, and exported to Stata version 17.0 statistical software for analysis. Bivariable and multivariable logistic regression analyses were employed. The *p*-value of <0.05 with 95% CI was used to declare statistical significance of association between YFS utilization and explanatory variables.

**Result:**

Less than one third (31.2%) of preparatory school students utilized YFS in Gambella town. Being married (AOR: 4.94, CI: 2.14, 11.38), having pocket money (AOR: 2.02, CI: 1.15, 3.56), no payment for YFS (AOR: 2.13, CI: 1.01, 4.50), having knowledge about YFS (AOR: 2.27, CI: 1.29, 4.00), convenient working time (AOR: 2.50, CI: 1.08, 5.83), and sexual experience (AOR: 3.38, CI: 1.90, 6.01) were the factors significantly associated with utilization of YFS in Gambella town.

**Conclusion:**

The study found that utilization of YFS in Gambella town was low. Being married, having pocket money, not asked payment for YFS, knowledge about YFS, convenient working time, and sexual experience were the factors positively affecting utilization of YFS in Gambella town. Therefore, the health decision makers better to design policies aimed to increase youths knowledge about YFS.

## Background

Youth-friendly services (YFS) are designed to meet the specific needs and preferences of young people, particularly adolescents. These services aim to provide a safe and supportive environment where young people can access information, advice, and treatment related to their physical, mental, and sexual health (1).

Utilization of YFS among school students is an important aspect of adolescents' healthcare. Adolescence is a critical period of development when physical, emotional, and social changes occurred. Young people may face various health issues such as sexual and reproductive health (SRH) concerns, mental health problems, substance abuse, and chronic diseases as well. Ensuring access to quality YFS is crucial to promote the overall well-being of adolescents (2–5).

Youths are not only at the healthiest period life, but they are also at critical periods faced set of threats to their health. They are also confronted with decisions leave them at risks of increasing morbidity and mortality. Moreover, they are at risk of unprotected sexual activity leading to Sexually Transmitted Infections (STIs), HIV/AIDS, unintended pregnancy, and unsafe abortions (6, 7).

Many of the diseases and injuries that affect adolescents are avoidable, yet they are often overlooked. They require government attention and sufficient funding. The burden of adolescent diseases varies widely around the world from 45% in Africa to 26% in South-East Asia, which are the home to 19% and 30%, respectively, of adolescents (6).

Ensuring adolescents SRH services is one of priorities in the 2030 sustainable development Goals (SDGs), assuring that every adolescent should have access to family planning, information, and education, and prevent forced marriages (8).

Globally there are more than 4.4 million young people between the age of 15 and19 years who have abortion every year and 40% of which is unsafe. The prevalence of Sexual transmitted Infections (STIs) including HIV/AIDS is relatively high among young people in Ethiopia (7, 9).

Adolescents in sub-Saharan Africa (SSA) had been influenced by various SRH problems including unsafe sexual practices which increased incidence of STIs and HIV, early pregnancy, and made them more susceptible to delivery complications (7, 10).

There is 13 million young aged girls of 15–19 years and girls younger than the age of 20 years have unintended births each year and the presence of unmet need for contraceptive exists among both married and unmarried women in the developing world. In this region, 41% and 15% of unsafe abortion takes place among young women age of 15–19 and 15–24 years respectively (9). There is an estimation of over 40% unintended pregnancy worldwide resulting from non-use, and ineffective use or method failure of contraceptive (11).

The well-being of women, men, and families was at risk due to unintended pregnancy, complications during pregnancy and childbirth, unsafe abortion, gender-based violence, STIs, including HIV, and problems with reproductive health (9). Adolescents who began sexual activity before they had the understanding and capacity to protect themselves were more likely to experience unplanned pregnancies, unsafe abortions, and sexually transmitted infections including HIV/AIDS (11).

The study conducted at University of Gondar stated the prevalence of STIs was 18.2%. We noticed that 46.6% of the female were sick. The most common STI signs and symptoms reported by 43.6 and 55.9% of male and female students, respectively, were genital ulcer and vaginal discharge (12). A history of early sexual activity was reported by 15% of adolescents. Likewise, 35.2% of adolescent females who had initiated sex previously had ever used alcohol. In the 832 adolescents who reported having sex, 11% (90 of them) reported having multiple partners (13).

Whether it was for essential health care or YFS, adolescents used SRH services. In the past year, only 37% of female and 39% of male adolescents received basic health services, while just 2% and 4% of both genders have ever used YFS (14). In the research area, YFS services were used by about 23.5% (95% CI: 20, 26.8) of in-school adolescents (64.2% of male students and 35.8% of female students) (15).

Additionally, age, marital status, place of residence, income, prior sexual activity, prior exposure to SRH information, prior discussion of YFS issues, perceived YFS risks, knowledge of the YFS facility and services, and distance from the health facility were all factors associated with the use of YFS services (15). Adolescents' use of YFS was found to be significantly associated with their sex, grade level, having pocket money for daily expenses, and parental education status (16).

According to the national study done by federal ministry of health on selected urban areas of Ethiopia, Southern Nations Nationalities and People region the finding showed that youth could not properly utilize the available health service (14). Moreover, to the best of authors knowledge there is no study conducted on YFS utilization and associated factors among preparatory school students in Gambella town despite high morbidity among high school students. Therefore, this study aimed to assess YFS utilization and its associated factors among preparatory school students in Gambella town, Southwest Ethiopia.

## Methods

### Study area, design and period

**Figure 1:**
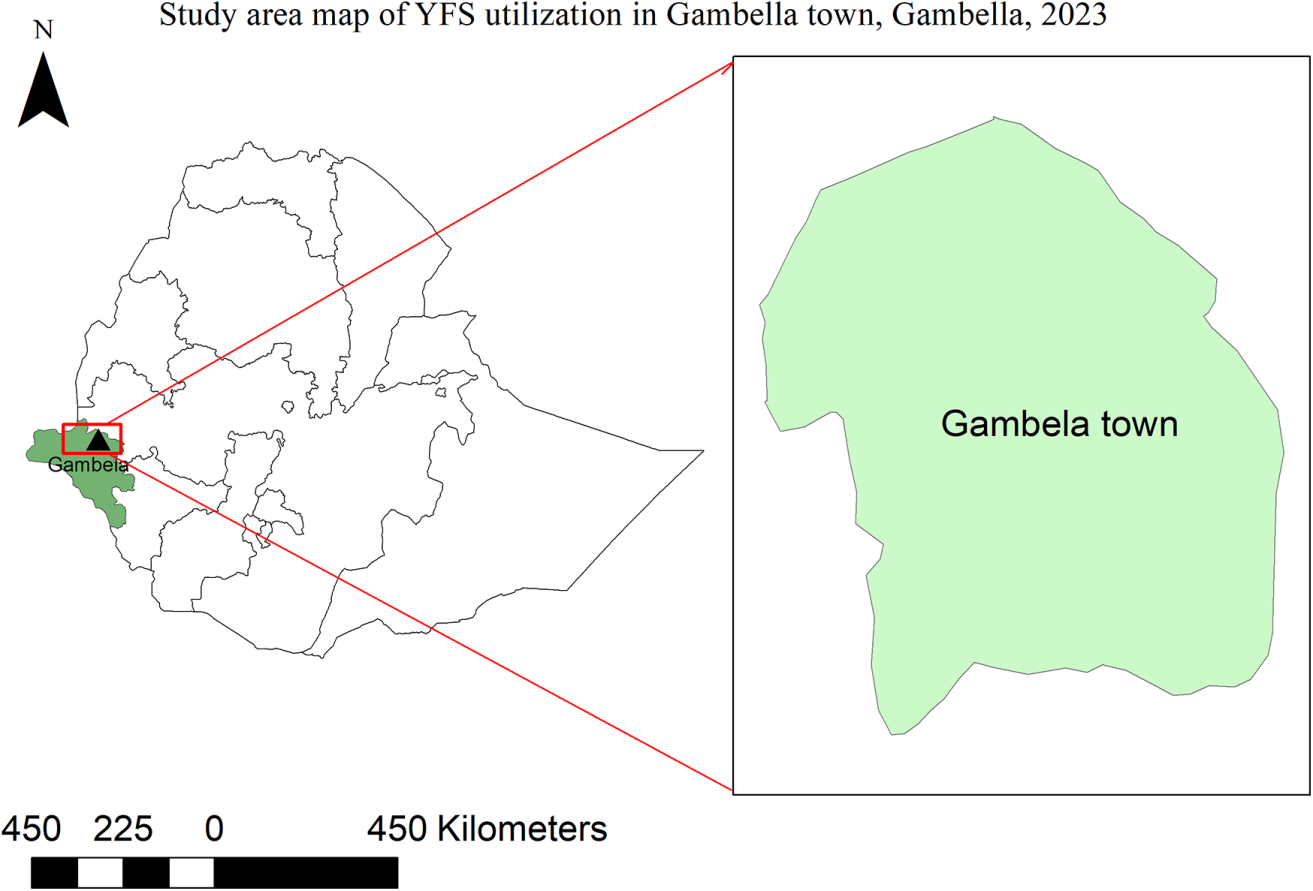
Map of study area, Gambella town, Gambella regional state, Southwest Ethiopia, 2023.

The study was conducted in Gambella town (Figure 1), capital of Gambella regional state, which is located 768 kilometers far from Addis Ababa, capital of Ethiopia. According to Gambella region Woreda base plan for 2023, the projected total population of the town is 67,451. There are two preparatory schools, Gambella and Elle preparatory schools. An institution based cross-sectional study was conducted among preparatory school students in Gambella town from June 01-30, 2023.

### Populations and eligibility criteria

All students enrolled for preparatory schools, grade 11 and 12, in Gambella town were the study populations for this study. The students aged between 15 and 24 years of Gambella and Elle preparatory schools were included in this study. Moreover, Students who were critically ill, absent during data collection, and unable to respond for mental health problems were excluded in the study.

### Sample size determination and sampling procedure

The sample size was estimated using single population proportion formula, assuming a 95% confidence level, a 5% margin of error, proportion (P) of youths utilizing YFS was 37% in Goba town (17).

$$n=\frac{Z(\alpha /2{)}^{2}*P(1\text{-}q)}{d^{2}}$$

Where *P* = 37%, margin of error =  0.05, and Z *α*/2 at 95% confidence level = 1.96. By taking the above values, the sample size was $n=\frac{{\left(1.96\right)}^{2}*(0.37)\;(1-0.63)}{{\left(0.05\right)}^{2}}=359$.

Adding 10 percent non-response rate the final sample size was 394.

**Figure 2:**
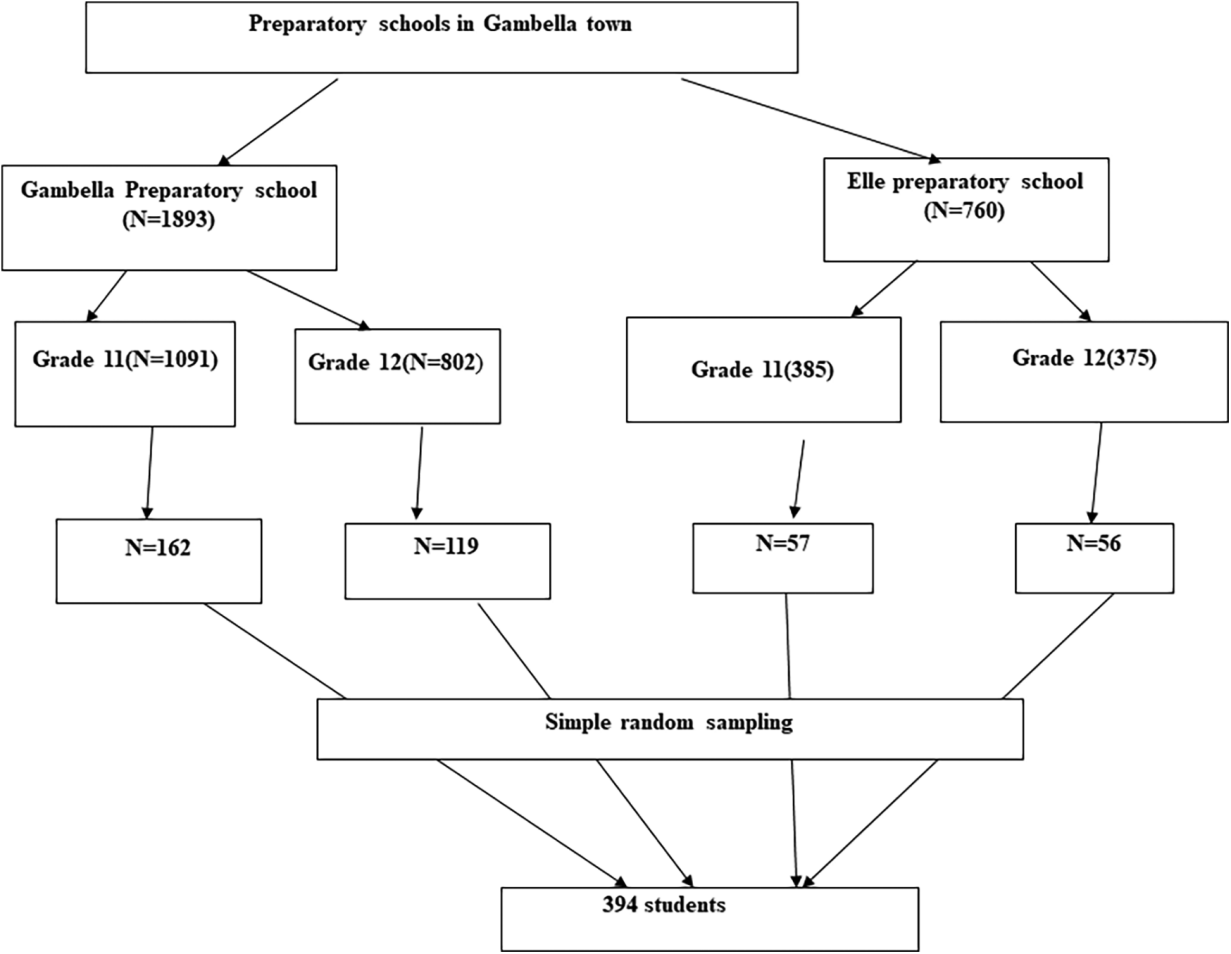
Pictorial depiction of sampling procedure, Gambella, Southwest Ethiopia, 2023.

Moreover, the study employed stratified sampling technique to select study participants. First we proportionally allocate the sample with their respective number of students and we randomly selected the students in each stratum and administered the questionnaire (Figure 2).

### Variables

**Outcome variable:** utilization of YFS was the outcome variable of the study.

#### Explanatory variables

The explanatory variables were grouped as sociodemographic variables (age, sex, residence, maternal and paternal educational level, marital status, and living arrangement), reproductive health and related variables (discussion with parents on reproductive health, and gain pocket money, sexual activity, age at first sex, number of sexual partners, condom use during sex, and knowledge of YFS), and health ]system related factors (distance from health facility, availability of YFS, convenient working time, and payment for YFS).

### Data collection procedures

The structured self-administered questionnaire was used to collect the data. The questionnaire was developed through consulting various previous literatures. The questionnaire comprises sociodemographic characteristics, sexual and reproductive health related factors, health system related factors, and knowledge-related factors.

Then the lists of students' identification cards were obtained from each school. The identification cards were randomly selected using a computer-generated simple random method. After all, the two BSc in midwifery graduate facilitators administered the questionnaire to the randomly selected students in each school.

### Operational definitions

#### Utilization YFS

It was classified as “**Yes**” if a student use at least one service from sexual and reproductive health service packages (medical checkup, consultations, FP counseling and methods, health education, VCT, STIs treatment, pregnancy test, safe abortion and other re-enforcement services rendered to the young people) at health facilities for last 12 months and classified as “**No**” otherwise (18).

#### Knowledge of YFS

It was measured through 6 questions. If the student answered the mean score and above, he/she was considered having good knowledge and poor knowledge otherwise (18).

### Data quality assurance

The structured questionnaire was prepared in English first and translated to local commonly spoken language (Amharic) for better understanding with respondents. Two facilitators who have bachelor degrees (Midwifery graduates) were employed, one facilitator for each school. Three days training was given for facilitators on the overall picture of questionnaires, how to facilitate the data collection and how to approach the respondents. Before actual data collection, pretesting on 5% (20 students) of the sample size was done at Abobo preparatory school. Close supervision of facilitators was done and data were checked for its completeness on daily basis.

### Data management and analysis

The collected data were checked for completeness. Then, data were coded, organized and entered into EpiData version 4.6 and exported to Stata version 17.0 for analysis. Descriptive statistical analysis (frequencies and percent), bivariable and multivariable logistic regressions were conducted. In bivariable logistic regression, variables having *p*-value of <0.2 with 95% CI were eligible for multivariable logistic regression. The overall goodness fit of binary logistic regression model was checked by Hosmer and Lemeshow test (*p*-value was ∼0.502). Assumptions of binary logistic regression such as multicollinearity and outliers were checked for the model. Adjusted Odds Ratio (AOR) with 95% CI was estimated to assess the strength of the association, and a *p*-value of <0.05 was used to declare statistically significant factors.

## Result

### Socio-demographic characteristics of study participants

A total of 394 preparatory school students were participated in this study. Of which, 270 (69.05%) were females and 294 (75.19%) of respondents were below 19 years old. Moreover, 365 (92.6%) were single, 29 (7.4), 169 (42.9%) were protestants, and 132 (33.2%) were Nuer regarding their ethnicity (Table 1).

**Table 1:** Percentage distribution of the study participants by socio-demographic characteristics, (*n* = 394).

Variable	Category	Frequency	Percent
Sex of respondent	Male	120	30.5
	Female	274	69.5
Age	15–19	296	75.1
	20–24	98	24.9
Marital status	Single	365	92.6
	Married	29	7.4
Grade	Grade 11	237	60.2
	Grade 12	157	39.8
Religion	Protestant	169	42.9
	Orthodox	72	18.3
	Catholic	88	22.3
	Missiles	26	6.6
	Others	39	9.9
Ethnicity	Anyuak	92	23.4
	Nuer	131	33.2
	Majangir	3	.8
	Amhara	50	12.7
	Oromo	118	29.9
Student live with	Both father and mother	240	60.9
	Father only	34	8.6
	Mother only	56	14.2
	Relatives/friends	53	13.5
	Alone	11	2.8
Father's education level	No education	81	20.6
	Primary	120	30.5
	Secondary	96	24.4
	Higher	97	24.6
Mother education level	No education	166	42.1
	Primary	136	34.5
	Secondary	44	11.2
	Higher	48	12.2

### Knowledge of family planning and STIs

About 300 (76.1%) students knew about family planning methods, where the most commonly known method was condoms (48.0%) followed by oral contraceptive pills (46.0%) and injectables (44.0%). Likewise, about 340 (86.7%) knew about STIs, where the most commonly known STI was HIV/AIDS (76.5%) followed by syphilis (28.8%) while others like gonorrhea and Chancroid are less known by 8.4% and 14.8% respectively (Table 2).

**Table 2:** Percentage distribution of the study participants by knowledge of family planning and STIs.

Student knowledge on family planning methods			
Variables	Category	Frequency	Percent
Know any method of family planning	Yes	300	76.1
	No	94	23.9
Know about condoms	Yes	144	48.0
	No	156	52.0
Know about implants	Yes	81	27.0
	No	219	73.0
Know about oral contraceptive pills	Yes	138	46.0
	No	162	54.0
Know about Injectables	Yes	132	44.0
	No	168	56.0
Know about IUCD	Yes	52	17.3
	No	248	82.7
Student knowledge of sexually transmitted infections (STIs)			
Ever heard about STIs	Yes	340	86.7
	No	52	13.3
know about syphilis	Yes	103	28.8
	No	255	71.2
Know about gonorrhea	Yes	30	8.4
	No	328	91.6
Know about Chancroid	Yes	53	14.8
	No	305	85.2
Know about HIV/AIDS	Yes	273	76.5
	No	84	23.5

### Knowledge of youth friendly services (YFS)

Among the total study participants, 325 (82.20%) had ever heard about YFS. The most commonly known services were family planning 160 (49.2%) followed by voluntary counseling and testing (VCT) for HIV 128 (39.4%) and abortion care 118 (36.3%). The major source of information for YFS were health care providers 191 (58.8%) followed by schools 150 (46.2%). Nearly about one third of the respondents, 130 (32.8%) had a parental discussion about reproductive health issues. The majority of students, 317 (80.5%), believed that youths have the rights to reproductive health services (Table 3).

**Table 3:** Percentage distribution of the study participants by knowledge of YFRH service and rights.

Variable	Category	Frequency	Percent
Information on Youth Friendly Reproductive Health Services			
Ever heard about YFS	Yes	325	82.2
	No	69	17.8
Know YFS provides FP services	Yes	160	49.2
	No	165	50.8
Know YFS provides HIV testing and counseling service	Yes	128	39.4
	No	197	60.6
Know YFS provides abortion care service?	Yes	118	36.3
	No	207	63.7
Know YFS provides STI treatment service	Yes	80	24.6
	No	245	75.4
Know YFS provides information and education service	Yes	76	23.4
	No	249	76.6
Know YFS provides condoms promotion service	Yes	100	30.8
	No	225	69.2
Sources of information about YFS			
Get the information about YFS from parents	Yes	42	12.9
	No	283	87.1
Get YFS information from schools	Yes	150	46.2
	No	175	53.8
Get YFS information from health providers	Yes	191	58.8
	No	134	41.2
Get YFS information from peers	Yes	102	31.4
	No	223	68.6
Get YFS information from media	Yes	60	18.5
	No	265	81.5
Ever discussed about RH issues with parents	Yes	130	32.8
	No	264	67.2
Youths have the right to have YFS	Yes	317	80.5
	No	77	19.5

### Sexual experience and protective behaviors

About 123 (31.2%) of the total participants had experienced sexual intercourse and more than half 75 (61.0%) of them experienced sex before age of 18 years. Among those who ever had sexual experience (*n* = 123), about 118 (95.9%) were currently sexually active. Nearly half, 57 (48.3%), had sexual intercourse with two sexual partners and about 8 (6.8%) had sex with three partners in the past 12 months. From sexually active students in the past 12 months (*n* = 118), only 43 (36.4%) of them (31 males out of 50 (62%) and 12 females out of 68 (17.6%) had used condoms during their last sexual intercourse. With regard to hormonal contraceptive methods use among sexually active female students (*n* = 68), about half, 35 (51.5%) of them had used hormonal contraceptive methods at last sexual intercourse in the past 12 months (Table 4).

**Table 4:** Percentage distribution of the study participants by sexual practice and protection use*.*

Variable	Category	Frequency	Percent
Ever had sexual intercourse	Yes	123	31.2
	No	271	68.8
Age at 1st sexual intercourse	<18 years	75	61.0
	≥18 years	48	39.0
Had sexual intercourse for last year	Yes	118	95.9
	No	5	4.1
Number of partner have sex for last year	Only one partner	53	44.9
	Two partners	57	48.3
	Three or more partner	8	6.8
Use condom during your last sex	Yes	43	36.4
	No	75	63.6
Use hormonal contraceptive during your last sex	Yes	35	51.5
	No	33	48.5

### Youth friendly services utilization

One hundred twenty-three (31.22%) of respondents said that they used at least one YFS in the past 12 months. About 69.1% of sexually active students had used at least one reproductive health service in the past 12 months. The most utilized service by sexually active students were family planning (71.8%) followed by VCT (24.7%). The major YFS providing facility utilized was family guidance association clinic (28.2%) followed by hospitals (27.1%) and youth center (23.5%). Among the service users, the majority (71.8%) were not asked to pay for the service they received (Table 5).

**Table 5:** Percentage distribution of preparatory school students by utilization of youth friendly services in gambella town (*n* = 394).

Variables	Category	Frequency	Percent
Used at least one YFS in last year	Yes	123	31.2
	No	271	68.8
Sexually active students used YFS las year	Yes	85	69.1
	No	38	30.9
Received FP services	Yes	61	71.8
	No	24	28.2
Received HIV testing and counseling services	Yes	21	24.7
	No	64	75.3
Received abortion care services	Yes	8	9.4
	No	77	90.6
Received STI treatment services	Yes	19	22.4
	No	66	77.6
Received condom promotion and provisions	Yes	23	27.1
	No	62	72.9
Place service received	Youth center	20	23.5
	Hospital	23	27.1
	Health center	18	21.2
	FGA clinic	24	28.2
Ever pay for YFS	Yes	24	28.2
	No	61	71.8

### Factors associated with YFS utilization

Multivariable analysis showed that being married, having a pocket money for daily expenses payment for YFS, convenience of working time, having knowledge about YFS, and having sexual intercourse were found to have statistically significant (*p*-value <0.05) factors with YFS utilization (Table 6). Married students were nearly five times more likely to use YFS than unmarried students (AOR: 4.95, 95% CI: 2.15, 11.42). Participants who gained a pocket money for daily expense were 2 times more likely to utilize YFS than those who did not gained pocket money for their daily expenses (AOR = 2.02, 95% CI: 1.15, 3.56).

**Table 6:** Multivariable analysis of factors affecting youth friendly service utilization among preparatory school students in gambella town.

Variables		YFS utilization		COR (95% CI)	AOR (95% CI)
		Yes	No		
Marital status	Married	39	12	9.75 (4.88, 19.47)	**4.94** (2.14, 11.38)*
	Single	85	255	1	1
Pocket money	Yes	56	52	3.40 (2.11, 5.64)	**2.02** (1.15, 3.56)*
	No	68	215	1	1
Discussion with parents	Yes	63	67	3.08 (1.92, 4.94)	1.64 (0.95, 2.84)
	No	61	200	1	1
YFS Availability	Yes	69	56	4.73 (2.90, 7.69)	1.77 (0.86, 3.63)
	No	55	211	1	1
Payment for YFS	No	46	27	5.24 (3.05, 8.99)	**2.13** (1.01, 4.50)*
	Yes	78	240	1	1
Working time	Convenient	27	15	4.68 (2.28, 9.85)	**2.5** (1.08, 5.83)*
	Inconvenient	97	252	1	1
Sexual experience	Yes	76	47	7.41 (4.59, 11.96)	**3.38** (1.90, 6.01)*
	No	48	220	1	1
Knowledge of YFS	Good	94	138	2.93 (1.82, 4.71)	**2.27** (1.29, 4.00)*
	Poor	30	129	1	1

YFS, youth friendly services; COR, crude odds ratio; AOR, adjusted odds ratio.

The bold AOR indicates significant category.

* Significant at *p* < 0.05.

The respondents who did not requested to pay for YFS were 2 times more likely to utilize youth friendly service utilization than those who use requested to pay for the service (AOR = 2.13, 95% CI: 1.01, 4.50). The respondents those working time were convenient for them were 2.51 times more likely to utilize youth friendly service utilization than those working time was not convenient for them (AOR = 2.51, 95% CI: 1.08, 5.83).

Moreover, students who experienced sexual intercourse were 3.38 times more likely to utilize YFS than those who did not experience sexual intercourse (AOR = 3.38, 95% CI: 1.90, 6.01). Students who had good knowledge about YFS were 2.27 times more likely utilized YFS than those who had poor knowledge (AOR = 2.27, 95% CI: 1.29, 4.01).

## Discussion

This study aimed to determine YFS utilization and determining factors among preparatory school students in Gambella town. The magnitude of YFS utilization among preparatory school students in Gambella town was found to be 31.73% (95% CI: 26.67%, 36.05%). Moreover, the study found that being married, gaining a pocket money, convenient working time, having knowledge about YFS, do not requested payment for YFS, and having sexual intercourse were the significant factors determining YFS utilization of preparatory school students in Gambella.

The rate of YFS utilization in this study was consistent with finding of studies conducted in North Shewa zone (32.70%), Amhara region (33.20%), Southwest Oromia (36.50%), Bahir Dar (32.20%), Debre Birhan (33.80%), Hadiya Zone (28.90%), and Debre Tabor town (28.80%) (7, 19–22). However, the finding was lower than findings of the studies conducted in Ethiopia in 2021 and in central Gondar zone which reported the magnitude of YFS utilization was 42.73%and 39.00%, respectively (23, 24). The potential reason for the inconsistency could be attributed to differences in socio-demographic factors and the time frame of the studies. Another plausible explanation could be the ongoing internal conflict and political instability in Ethiopia since 2021. The conflict and instability may have had an impact on the overall accessibility and availability of YFS throughout Ethiopia, with particular implications for the study region of Gambella (3–5).

Moreover, this study finding showed that marital status was significantly associated with YFS. Married students were about five times more likely to use YFS than unmarried students. This finding was in line with the study conducted in Bale zone, Oromia region (19). Likewise, the study revealed that pocket money was significantly associated with YFS utilization. Students who gained a pocket money were twofold more likely to utilize YFS. This finding was supported by the study conducted in Aleta Wondo town, southern Ethiopia (25). Furthermore, this study showed that payment for the service was significantly associated with YFS utilization. The respondents who did not requested to pay for YFS were more likely to utilize YFS than those who use requested to pay for the service. This finding was also supported by finding reported from Nepal (26).

The finding showed that working time was significantly associated with YFS utilization. The respondents those working time were convenient for them were 2.51 times more likely to utilize YFS utilization than those working time was not convenient for them (23).

According to this study's finding, sexual intercourse was significantly associated with YFS utilization. Students who experienced sexual intercourse were about 3 times more likely to utilize YFS utilization than those who did not experience sexual intercourse.

Study revealed that having knowledge about YFS was significantly associated with YFS utilization. Students who had good knowledge about YFS were 2 times more likely utilized youth-friendly reproductive health services than those who had poor knowledge (AOR = 2.27, 95% CI: 1.29, 4.01). The finding of the study was supported by study conducted in Areka Town, Sothern Ethiopia (18).

### Policy and practical implications

Policies should focus on increasing awareness and integrating comprehensive sexual and reproductive health education into school curriculums. Financial barriers should be eliminated by making YFS free or affordable. Services should be available at convenient times to align with students' schedules. Practically, promoting YFS through schools and community centers, training healthcare providers to be youth-friendly, and encouraging parental involvement are essential. Moreover, regular monitoring and evaluation will help assess the effectiveness of these services and make necessary improvements.

## Conclusion

The study revealed that utilization of YFS in Gambella town was lower than the national and international target. Being married, the students having pocket money, the students not asked payment for YFS, having knowledge about YFS, convenient working time, and having sexual experience were the factors positively affecting utilization of YFS in Gambella town. Therefore, the health decision makers and health planners better to design policies aimed to increase youth's knowledge about YFS, make working time convenient, and make YFS services free and easily accessible for youths.

## Data Availability

The original contributions presented in the study are included in the article/Supplementary Material, further inquiries can be directed to the corresponding author.
